# Intussusception in Premature Baby: Unusual Cause of Bowel Obstruction and Perforation

**DOI:** 10.21699/jns.v6i1.407

**Published:** 2017-01-01

**Authors:** Kanokkan Tepmalai, Thanyaluck Naowapan, Jesda Singhavejsakul, Mongkol Laohapensang, Jiraporn Khorana

**Affiliations:** 1 Division of Pediatric Surgery, Department of Surgery, Faculty of Medicine, Chiang Mai University Hospital, Chiang Mai University, Thailand; 2 Division of Pediatric Surgery, Department of Surgery, Faculty of Medicine, Siriraj Hospital Mahidol University, Thailand

**Keywords:** Premature, Necrotizing enterocolitis, Neonate

## Abstract

Intussusception in a premature baby is a rare condition. We report a male preterm infant, who developed abdominal distension and abdominal wall erythema. He was operated with suspicion of NEC but an ileo-ileal intussusception and intestinal perforation were encountered at operation.

## CASE REPORT

A male preterm infant, weighing 1190 grams, was delivered by Cesarean section at 29 weeks of gestation secondary to severe maternal preeclampsia with HELLP syndrome. The Apgar score was 1 at 1 minute. The infant required assisted ventilation immediately after birth with no spontaneous respiration and severe birth asphyxia. Surfactant replacement therapy was administered and umbilical venous and arterial catheters were inserted. Total parenteral nutrition was initiated at one day of age and orogastric feeding with breast milk was started on day two. His general condition remained stable until four days of age. The infant developed bilious vomiting with coffee-ground content and progressive abdominal distension. He passed a small amount of true meconium daily. The plain abdominal radiograph showed generalized bowel dilatation without pneumatosis intestinalis or free air. Necrotizing enterocolitis (NEC) was suspected. Feedings were discontinued on day 4. The umbilical catheters were removed. Broad-spectrum antibiotics (Meropenem and Metronidazole) were given while closely monitoring the patient’s condition. Two days after conservative management, the patient had marked abdominal distension with erythema at the right abdominal wall. Immediate abdominal radiography showed pneumoperitoneum (Fig.1). At surgery, an ileo-ileal intussusception at 58 cm, with proximal perforations at 52 cm from the ligament of Treitz, were identified (Fig.2). The 27centimeter long necrotic segment was resected and a diverting double-barrel ileostomy was created due to the inflammation of the remaining bowel. The total remaining small bowel length was 50 cm. The postoperative course was uneventful. The infant’s condition improved and the ileum was re-anastomosed after six weeks. The pathology report showed transmural infarction and perforation of ileum without evidence of a leading point.

**Figure:**
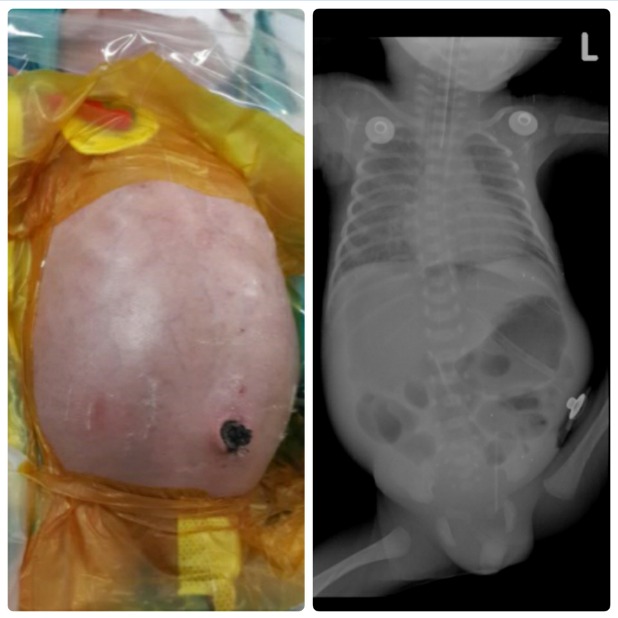
Figure 1: The abdominal characteristics and the radiograph of the baby at six days of age with distension of the abdomen and pneumoperitoneum.

**Figure:**
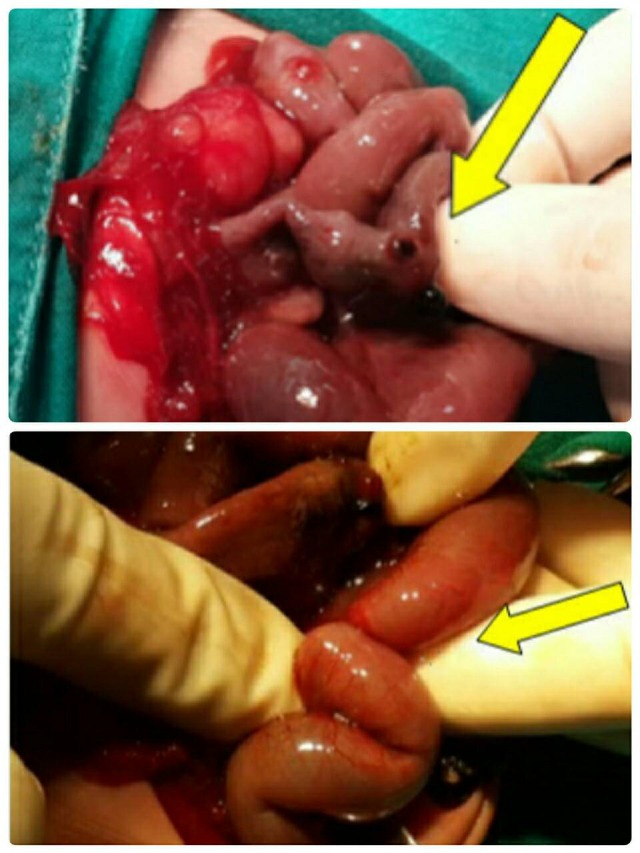
Figure 2: Perforation Site (arrow, upper frame) and Ileo-leal Intussusception (arrow, lower frame)

## DISCUSSION

Intussusception in the neonate is extremely rare. Only less than 50 cases have been reported in literature.[1-5] Clinical signs of intussusception appeared to be nonspecific. Clinical features of intussusception and NEC appear to be very similar. Most of the patients in the reported cases had abdominal distension, bilious vomiting and bloody stool which are found together in NEC. More than half of premature neonates with intussusception were diagnosed preoperatively with NEC. [6-8]

The etiology of neonatal intussusception remained unclear. Ueki, 2005 proposed neonatal intussusception associated with hypoxia.[2] The association of intussusception with intestinal atresia is well known in the full-term neonate. Some reviews [9] suggest that intussusception may occur first, presumably prenatally, leading to vascular impairment with subsequent bowel necrosis resulting in secondary congenital intestinal atresia, but this association is not evident in preterm infants. Preterm intussusception may be associated with a meconium plug, abdominal intervention or concomitant necrotizing enterocolitis.[3]

Radiological signs may be helpful in distinguishing between intussusception and NEC. In intussusception, signs of obstruction are evident such as dilatation of bowel loops and occasionally gas-fluid levels, whereas the hallmark of NEC is pneumatosis intestinalis with generalized bowel dilatation. Pneumoperitoneum can be found in 20% of cases.

The recommended treatment is resection and anastomosis if manual reduction is not possible. In selected cases with unhealthy bowel or unstable vital signs, resection and ostomy can be performed. Our case had severe intra-abdominal contamination and the remaining bowel was inflamed.

In conclusion, there are no risk factors to differentiate between intussusception and NEC. Intussusception is easily misdiagnosed as NEC especially in preterm infants. The treatment of each condition is different. Intussusception requires early surgical intervention to prevent bowel necrosis, whereas surgical intervention is often withheld in NEC. If a patient with suggested NEC had a poor clinical response to medical treatment and abdominal radiograph suggestive of small bowel obstruction, intussusception should be suspected. Ultrasonography facilitated an early diagnosis in suspected cases. Surgical intervention should be performed with a good outcome after resection of the compromised bowel.

## Footnotes

**Source of Support:** Nil

**Conflict of Interest:** Nil
